# LONGITUDINAL ASSOCIATION BETWEEN HOSPITALIZATION AND TRANSFER TO RESIDENTIAL CARE FACILITIES AMONG OLDER ADULTS

**DOI:** 10.1093/geroni/igac059.1974

**Published:** 2022-12-20

**Authors:** Reza Amini, Amat Sidhu

**Affiliations:** University of Michigan-Flint, Ann Arbor, Michigan, United States; University of Michigan-Flint, Flint, Michigan, United States

## Abstract

Dementia increases the risk of post-hospitalization transfer to residential care facilities (RCF), increasing mortality risk. The risk of falling is significantly higher among people with dementia, leading to more hospital admissions (HA). This study examines the association between HA and transferring to RCF controlling for dementia and falling. This secondary data analysis used nine years of data from the National Health and Aging Trends Study (2011-2019) with 2,548 participants (each wave). Transfer to RCF was measured based on the residency status, HA and history of falling by survey questions, and dementia (probable, possible, no dementia) by combining Clock Drawing Test, word recall test, and previous dementia diagnosis. Falling increased the risk of transition to RCF by 31%; however, this association was not statistically significant. Dementia was a significant predictor for transition to RCF (Odds Ratio (OR)=3.889). Transferring to RCF was significantly associated with HA (OR=1.835); after including falling, OR increased to 2.067, showing falling moderates this association. Including dementia into the model dropped HA’s OR to 1.620. The interaction between falling and dementia showed that frequent HA could increase the risk of transition to RCF by 61%. Probable dementia plus a history of falling can increase the risk of transition to RCF by 22 times and 14 times for those cases of probable dementia without a history of falling. These results may reflect the necessity of screening for the risk of falling and dementia among community-dwelling older adults to prevent falling, hence, frequent HA and ultimately transition to RCF.

